# Architecture distortion score (ADS) in malignancy risk stratification of architecture distortion on contrast-enhanced digital mammography

**DOI:** 10.1007/s00330-020-07395-3

**Published:** 2020-10-30

**Authors:** Yonggeng Goh, Ching Wan Chan, Premilla Pillay, Herng-Sheng Lee, Huay-Ben Pan, Bao-Hui Hung, Swee Tian Quek, Chen-Pin Chou

**Affiliations:** 1 Department of Diagnostic Imaging, National University Hospital, Singapore, Singapore; 2 Department of Breast Surgery, National University Hospital, Singapore, Singapore; 3 Department of Pathology and Laboratory Medicine, Kaohsiung Veterans General Hospital, Kaohsiung, Taiwan; 4 Department of Radiology, Kaohsiung Veterans General Hospital, Kaohsiung, Taiwan; 5 Department of Medical Laboratory Sciences and Biotechnology, Fooyin University, Kaohsiung, Taiwan

**Keywords:** Contrast-enhanced digital mammography, Architecture distortion

## Abstract

**Objective:**

To develop a risk predictor model in evaluation of tomosynthesis-detected architectural distortion (AD) based on characteristics of contrast-enhanced digital mammography (CEDM).

**Methods:**

Ninety-four AD lesions on CEDM in combination with tomosynthesis were retrospectively reviewed from 92 consecutive women (mean age, 52.4 years ± 7.9) with abnormal diagnostic or screening mammography. CEDM results were correlated with histology of ADs using cross-tabulation for statistical analysis. Predictors for risk of malignancy from CEDM characteristics (background parenchyma enhancement, degree of AD enhancement, enhancing morphology, size of enhancement, and enhancing spiculations) and patient’s age were evaluated using logistic regression. We propose a sum score, termed AD score (ADS), for risk stratification and corresponding suggested BI-RADS category.

**Results:**

Thirty-three of ninety-four (35.1%) of detected AD lesions were malignant. The sensitivity, specificity, PPV, and NPV of CEDM in evaluation of malignant AD are 100%, 42.6%, 48.5%, and 100%, respectively. Absence of AD enhancement on CEDM is highly indicative of no underlying malignancy. On multivariate analysis, the predictors on CEDM with statistical significance are (1) marked intensity of AD enhancement (OR, 22.6; 95%CI 3.1, 166.6; *p* = .002); and (2) presence of enhancing spiculations (OR, 9.1; 95%CI 2.2, 36.5; *p* = .002). A prediction model whose scores (ADS) given by ranking of OR of all predictors with AUC of 0.934 and Brier score of 0.0956 was developed.

**Conclusion:**

ADS-based lesion characterization on CEDM enables risk assessment of tomosynthesis-detected AD lesions.

**Key Points:**

*• Architecture distortions presenting with marked enhancement intensity and presence of enhancing spiculations are highly associated with risk of malignancy.*

*• Absence of architecture distortion enhancement in minimal or mild background parenchyma enhancement on CEDM indicates low risk of breast malignancy (NPV = 100%).*

**Electronic supplementary material:**

The online version of this article (10.1007/s00330-020-07395-3) contains supplementary material, which is available to authorized users.

## Introduction

Architecture distortion (AD) is the third most common abnormality detected on mammograms [1, 2] and is defined as “distortion of the breast parenchymal architecture without a definable mass” according to the Breast Imaging Reporting and Data System (BI-RADS) [3]. There are multiple causes of AD, ranging from breast malignancies to a variety of benign causes such as trauma, post-operative changes, complex sclerosing lesions, or radial scar [4]. Primary AD (defined as cases without history of trauma, intervention, or infection) has been described as a common presentation of non-palpable breast cancer [5]. However, there is conflicting data in the literature regarding its risk of malignancy with positive predictive values (PPVs) ranging from as low as 10% to as high as 83% [1, 2, 5–7]. In the absence of a non-invasive modality to accurately differentiate between benign and malignant causes of AD, an invasive procedure such as biopsy or surgical excision is often required. This hence poses a diagnostic and management challenge to breast radiologists and surgeons. In addition, this issue is expected to worsen due to the increased detection of AD from the growing clinical use of digital breast tomosynthesis (DBT) [6–8].

Therefore, several studies have proposed the use of a contrast-enhanced modality such as magnetic resonance imaging (MRI) to evaluate primary AD [9–11]. This is based on the hypothesis that malignant causes of AD will demonstrate increased enhancement secondary to angiogenesis, thereby differentiating it from benign causes. In these studies, absence of enhancement of AD correlate on MRI has been shown to be reassuring with high negative predictive values (NPVs) of 80–100%. However, the high costs and general long waiting times for breast MRI may render it non-feasible for many healthcare settings and institutions in evaluation of all AD cases.

Contrast-enhanced digital mammography (CEDM) is a new and emerging breast imaging modality which uses contrast media and the principle of dual-energy subtraction to evaluate enhancement of breast lesions. CEDM is less expensive compared to MRI and has demonstrated results comparable to MRI in many settings of breast imaging such as lesion characterization, local staging, and evaluating response to neoadjuvant therapy [12–16]. CEDM, as a cost-effective substitute, could evaluate AD in a way similar to MRI and provides a perfect correlation of mammographic and contrast-enhanced findings. However, there is a lack of literature to support its use. As far as we know, there is only a single study published to date and the sample size was small [17].

Hence, this study, with a larger sample size is designed to investigate the diagnostic capabilities of CEDM in the evaluation of AD detected on DBT, in the hope to potentially reduce the number and need for biopsy or surgical excision in patients with benign causes of AD. In this study, we also aim to develop a practical scoring model to stratify the malignant risk of AD on CEDM based on each imaging characteristic.

## Materials and methods

This retrospective Health Insurance Portability and Accountability Act–compliant review was approved by our institutional review board. The need for informed consent was waived.

### Patient selection

Retrospective study of radiology database identified 700 consecutive CEDM examinations in combination with tomosynthesis for abnormal diagnostic or screening imaging findings at the Department of Radiology, Kaohsiung Veterans General Hospital, from February 2012 to Nov 2019. The important findings (mass, calcifications, focal asymmetry, AD) on FFDM (full-field digital mammography) and DBT were based on independent review of three board-certified breast radiologists (C.C.P., H.B.P., and B.H.H.) trained on breast imaging with 17, 27, and 5 years of experience. AD detected on FFDM and DBT was achieved when at least two out of three radiologists had the same imaging report of AD. Lesions seen at both FFDM and DBT were considered to be FFDM detected. Results of CEDM for all detected ADs were correlated with histopathological findings from biopsy or surgical excision results. Patients who did not undergo biopsy or surgery due to technique limitations or other reasons would require a minimum imaging follow-up (DBT and breast MRI) of 2 years to ascertain true benign status. The exclusion criteria were (1) patients lacking biopsy proof and 2 years of DBT follow-up; (2) ADs secondary to previous surgery.

### CEDM image acquisition protocol

All CEDM images were acquired using a mammography system (Selenia Dimensions, Hologic) with dual-energy exposure. The contrast medium Omnipaque 350 (GE Healthcare Inc.) was injected into patients via an automatic power injector (Vistron CT injection system, Medrad) at a volume of 1.5 ml/kg of body weight and rate of 3 ml/s through a peripheral intravenous cannula. After completion, patients were disconnected from the automatic power injector.

The CEDM images were obtained starting at 2 min after contrast medium injection. Mediolateral oblique (MLO) and craniocaudal (CC) views of the breast with the lesion of concern would be obtained first followed by CC and MLO views of the contralateral breast. Two exposures (i.e., high-energy at 45–49 kVp and low-energy beam at 26–32 kVp) were obtained almost simultaneously for each view and a subtracted image between the two was generated to visualize contrast enhancement of both breasts. The image acquisition of all 4 views was completed within 7 min.

### CEDM image interpretation and analysis

**Fig. 1:**
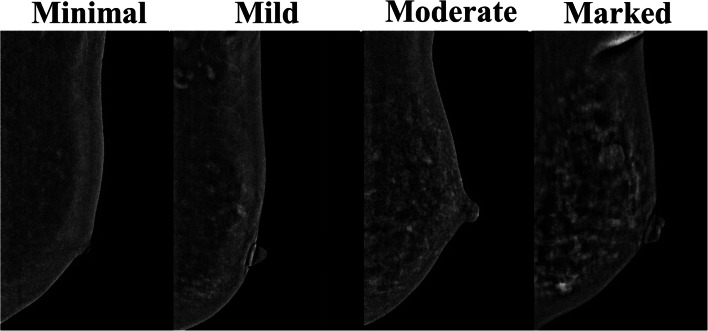
Representative contrast-enhanced digital mammography (CEDM) show breasts with minimal, mild, moderate, and marked background parenchyma enhancement (BPE)

The subtracted CEDM images were reviewed by 3 breast radiologists (C.C.P., H.B.P., and B.H.H.) who were blinded to final histology results. Consensus expert opinion was taken to be the agreement of at least 2 of 3 of the participating radiologists. Firstly, the background parenchymal enhancement (BPE) of both breasts was evaluated and categorized as follows: (1) minimal; (2) mild; (3) moderate; or (4) marked (Fig. 1).

**Fig. 2:**
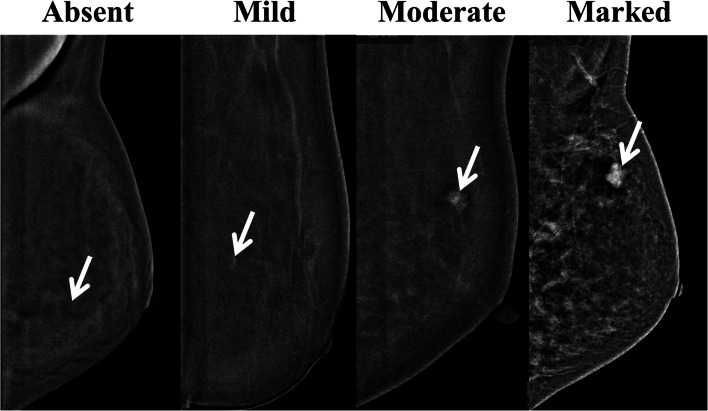
A standard reference image for degree of common lesion enhancement on contrast-enhanced digital mammography (CEDM) with absent, mild, moderate, and marked

Secondly, the enhancement characteristics of AD were evaluated. These are based on characteristics such as (1) enhancement intensity; (2) size; (3) morphology; and (4) margins. For intensity of contrast enhancement, the lesions were graded as follows: (1) absent; (2) mild; (3) moderate; or (4) marked using our standard reference image for CEDM enhancement (Fig. 2). The sizes of the AD enhancement based on only CEDM images were measured using a three-monitor workstation (SecurViewDx, Hologic) capable of displaying full-field digital mammography (FFDM), DBT, and CEDM. Characteristics of non-contrast mammographic images were not used for data analysis.

**Fig. 3:**
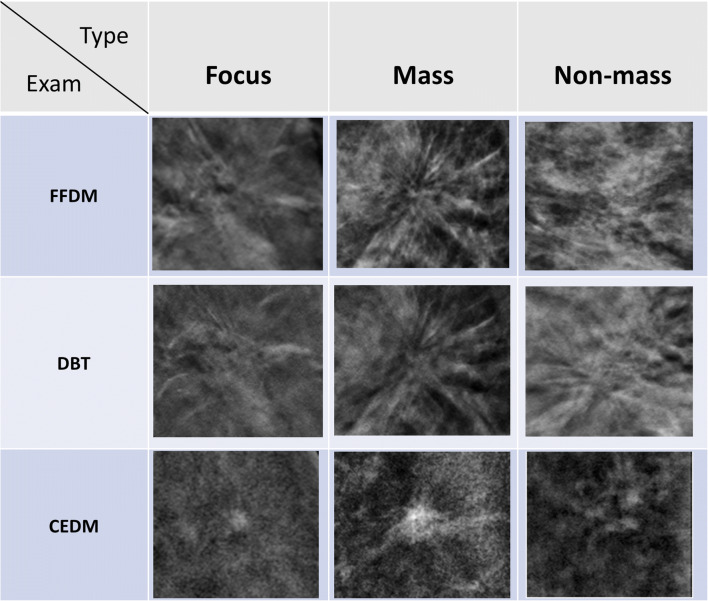
Images of AD lesions at full-field digital mammography (FFDM), digital breast tomosynthesis (DBT), and contrast-enhanced digital mammography (CEDM). AD enhancing pattens at CEDM are classified into three groups: focus (< 5 mm), mass with and without enhancing spiculations, and non-mass

The morphology of enhancement was categorized as follows: (1) focus (< 5 mm); (2) mass; or (3) non-mass enhancement according to lexicons as described for breast MRI developed by the American College of Radiology [3] (Fig. 3). Lastly, the AD lesions which demonstrated mass or non-mass enhancement were assessed for the presence or absence of enhancing spiculations (Fig. 3).

### Data and statistical analysis

The presence or absence of AD enhancement on CEDM was compared to histological findings obtained from available biopsy or surgical excision reports in a categorical format (i.e., benign or malignant). In cases where no histological correlation was observed, patients were followed up for the next 2 years to confirm its true benign status. Cross-tabulation was performed to evaluate the overall diagnostic accuracy, sensitivity, specificity, PPV, and NPV of CEDM in evaluation of AD. In this study, surgical results of high-risk lesions such as atypical ductal hyperplasia (ADH) and lobular carcinoma in situ (LCIS) were classified as benign cases.

Predictors for AD from CEDM characteristics (i.e., BPE, intensity of contrast enhancement, enhancing morphology, size of enhancement, and enhancing spiculations), patient’s age, and risks of malignancy were also evaluated using univariate and multivariate logistic regression analyses. Odds ratios (OR) with 95% confidence intervals (CI) were presented. Receiver operating characteristic curve (ROC) analysis and Youden index were performed for size of AD lesion enhancement on CEDM to determine the optimal cutoff value for maximizing specificity and sensitivity. Prediction models for AD were developed. A sum score, termed AD score (ADS), was derived from the prediction model for risk stratification.

All the statistical analyses were performed by using the SPSS software (Version 22; PASW Statistics) with statistical significance set at *p* < 0.05.

## Results

A total of 851 target lesions in 700 women were noted in our CEDM database. After excluding 753 mammographic lesions (mass, calcifications, focal asymmetry, and negative findings) other than AD and 4 lesions with incomplete imaging exams, there were a total of 94 AD lesions (61 benign, 33 malignant) detected from 92 women (1 patient had 3 lesions). Mean age of the enrolled patients was 52.4 ± 7.9 (SD) years. The 33 breast malignancies were confirmed by histopathological diagnosis and followed by surgical management. Fifty-one (83.6%) of 61 benign lesions had histopathological correlation with imaging-guided biopsy (ultrasound, tomosynthesis, MRI) or surgical excision after wire localization. The remaining 10 (16.4%) lesions with no histological correlation were followed up closely with DBT for at least 2 years to ascertain true benign status. There were no patients excluded from this study due to previous surgery or due to the lack of follow-up/surgery. Twenty-one (22.3%) of 94 AD lesions were detected on DBT only. Patient characteristics and histopathology of AD lesions included in the analysis are shown in Table 1.

**Table 1:** Patient characteristics and histopathology results of 94 AD lesions

Parameter	Value
Age (years)	
Mean ± SD	52.4 ± 7.9
Range	30–69
Breast density category	
Almost entirely fatty	2 (2%)
Scattered densities	2 (2%)
Heterogeneous dense	79 (84%)
Extremely dense	11 (12%)
Size of enhancing AD (cm)	
Mean ± SD	1.06 ± 1.13
Range	0.1–6.2
Malignancy (*n* = 33)	
Invasive ductal carcinoma	18 (54.5%)
Ductal carcinoma in situ	10 (30.3%)
Invasive lobular carcinoma	4 (12.1%)
Tubular carcinoma	1 (3.0%)
Benign disease (*n* = 61)	
Fibrocystic change with stromal fibrosis	23 (37.7%)
Radial scar	17 (27.9%)
Benign by imaging follow-up ≥ 2 years	10 (16.4%)
Sclerosing adenosis	6 (9.8%)
High-risk lesions (ADH or LCIS)	5 (8.2%)

*AD* architectural distortion, *SD* standard deviation, *ADH* atypical ductal hyperplasia, *LCIS* lobular carcinoma in situ

### Benign lesions

Fibrocystic change with stromal fibrosis (23/61, 37.7%) forms the majority of benign lesions in our study. Of the 17 radial scars, 6 were diagnosed via direct surgical excision after wire localization with no evidence of disease upgrade after surgery. The remaining 11 radial scar lesions were diagnosed via a combination of DBT-guided VABB (vacuum-assisted breast biopsy) and negative DBT results on follow-up (mean, 30 months; range, 11–85 months). Ten lesions with no histological diagnoses were diagnosed as benign on additional breast MRI or on follow-up imaging. In these 10 patients, 6 underwent breast MRI for very subtle lesions on both DBT and CEDM. The final assessments of these 6 lesions were regarded as BI-RADS category 1–2 on the basis of MRI findings. The remaining 4 of 10 patients had small AD lesions with no suspicious enhancement on CEDM. These were highly indicative of no underlying malignancy and would have been difficult for tomosynthesis-guided biopsy. Nonetheless, all 10 lesions were followed up for at least 2 consecutive years with DBT and ultrasound to affirm the absence of malignancy.

### High-risk lesions

High-risk lesions (5/61) (i.e., atypical ductal hyperplasia (*n* = 2), lobular carcinoma in situ (*n* = 2), flat epithelial atypia (FEA) (*n* = 1)) were considered benign in our study and made up the minority (8.2%) of benign lesions. All 5 lesions were diagnosed via VABB and subsequent surgical excisions. High-risk lesions would be re-classified as malignant if lesions exhibit pleomorphism or evidence of disease upgrade upon excision. However, none of the 5 high-risk lesions showed disease upgrade on excision and was classified as benign in this study.

### CEDM characteristics

The following CEDM characteristics were collected as variables during data analysis, namely (1) CEDM BPE; (2) size of AD on CEDM; (3) CEDM enhancement morphology; (4) AD enhancement intensity; and (5) presence/absence of enhancing spiculation on CEDM. Cross-tabulation was performed for each of these variables to determine the sensitivity, specificity, PPV, and NPV of CEDM in evaluation of AD. In this study, the variables which demonstrate the highest PPV for malignancy on CEDM are as follows: (1) marked enhancement intensity of AD lesion on CEDM (PPV = 89.5% (17/19)); (2) presence of enhancing spiculations (PPV = 70.6% (24/34)); (3) mass/non-mass enhancing morphology on CEDM (PPV = 55.6% (30/54)). These results are summarized in Table 2.

**Table 2:** Cross-tabulation table of CEDM characteristics with histopathology (*N* = 94)

		Histology		PPV (%)
		Benign	Malignant	
Background parenchyma enhancement (BPE)	Minimal/mild	53	26	32.9 (26/79)
	Moderate/marked	8	7	46.7 (7/15)
Size of AD enhancement	< 0.7 CM	37	4	9.8 (4/41)
	≥ 0.7 CM	24	29	54.7 (29/53)
Morphology	Absent	26	0	0
	Focus	11	3	21.4 (3/14)
	Mass/non-mass	24	30	55.6 (30/54)
AD enhancement intensity	Absent/mild	47	7	13.0 (7/54)
	Moderate	12	9	42.9 (9/21)
	Marked	2	17	89.5 (17/19)
Enhancing spiculations	Present	10	24	70.6 (24/34)
	Absent	51	9	15.0 (9/60)
CEDM enhancement	Absent	26	0	0
	Present	35	33	48.5 (33/68)
	Overall AD evaluation with CEDM (*N* = 94)			
	Result	95% CI (lower)	95% CI (upper)	
Sensitivity	100% (33/33)	89.4%	100%	
Specificity	42.6% (26/61)	30.0%	55.9%	
PPV	48.5% (33/68)	36.2%	61.0%	
NPV	100% (26/26)	86.8%	100%	
Accuracy	62.8% (59/94)	52.2%	72.5%	

### Statistical analysis and prediction model

**Fig. 4:**
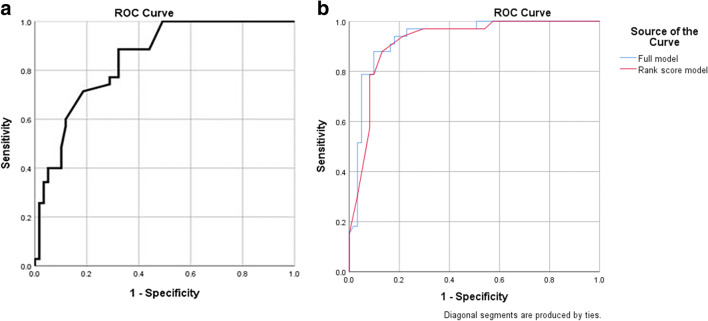
(**a**) ROC curve for enhancing size of AD on CEDM demonstrates an optimal cutoff value of approximately 0.7 cm (sensitivity 88.6%; specificity, 67.8%). (**b**) ROC curves for both full model and prediction model based on the ranking of the odds ratios (i.e., ADS (architecture distortion score) on CEDM show no statistical difference between the two)

Overall, the sensitivity, specificity, PPV, and NPV of CEDM in the evaluation of AD are 100% (95%CI 89.4%, 100%), 42.6% (95%CI 30.0%, 55.9%), 48.5% (95%CI 36.2%, 61.0%), and 100% (95%CI 86.8%, 100%), respectively (Table 2). A ROC curve analysis (Fig. 4) for AD size on CEDM was performed which demonstrated an optimal cutoff size for enhancing AD lesion on CEDM to be approximately 0.70 cm (sensitivity, 88.6%; specificity, 67.8%).

At univariate analysis, the following features on CEDM were shown to demonstrate a significant association with breast malignancy: (1) size of contrast enhancement (≥ 0.7 cm); (2) enhancing morphology (mass/non-mass enhancement); (3) enhancement intensity (moderate to marked); and (4) presence of enhancing spiculations. However, multivariate analysis showed that breast malignancy was significantly associated with (1) marked intensity of AD lesion on CEDM (OR 22.6; 95%CI 3.1, 166.6; *p* = .002); and (2) presence of enhancing spiculations (OR 9.1; 95%CI 2.2, 36.5; *p* = .002). Findings are summarized in Table 3.

**Table 3:** Association between breast malignancy and AD characteristic on CEDM

	Histopathology		Unadjusted analysis		Adjusted analysis	
Characteristic	Benign	Malignant	Odds ratio (95% CI)	*p* value	Odds ratio (95% CI)	*p* value
Mean age (SD)	52.1 (7.1)	52.8 (9.3)	1.01 (0.96–1.07)	0.692	1.05 (0.96–1.14)	0.279
Background parenchyma enhancement (BPE)						
- Minimal + mild	53 (67.1%)	26 (32.9%)	1.0	…	1.0	…
- Moderate + marked	8 (53.3%)	7 (46.7%)	1.8 (0.6–5.5)	0.310	2.4 (0.4–13.9)	0.315
Size of AD enhancement						
- < 0.7 cm	37 (90.2%)	4 (9.8%)	1.0	…	1.0	…
- ≥ 0.7 cm	24 (45.3%)	29 (54.7%)	11.2 (3.5–35.8)	< 0.001***	1.5 (0.1–15.5)	0.738
Enhancing morphology						
- Absent	26 (100%)	0 (0%)	NA	NA	NA	NA
- Focus	11 (78.6%)	3 (21.4%)	1.0	…	1.0	…
- Mass/non-mass	24 (44.4%)	30 (55.6%)	4.6 (1.1–18.3)	0.031*	1.8 (0.2–18.5)	0.638
Enhancement intensity of AD						
- Absent + mild	47 (87.0%)	7 (13.0%)	1.0	…	1.0	…
- Moderate	12 (57.1%)	9 (42.9%)	5.0 (1.6–16.3)	0.007**	1.4 (0.3–6.5)	0.696
- Marked	2 (10.5%)	17 (89.5%)	57.1 (10.8–302.1)	< 0.001***	22.6 (3.1–166.6)	0.002**
Enhancing spiculations of AD						
- Present	10 (29.4%)	24 (70.6%)	13.6 (4.9–37.8)	< 0.001***	9.1 (2.2–36.5)	0.002**
- Absent	51 (85.0%)	9 (15.0%)	1.0	…	1.0	…

*AD* architectural distortion, *CEDM* contrast-enhanced digital mammography, *CI* confidence interval, *NA* not available, *SD* standard deviation

*p < 0.05, **p < 0.01, ***p < 0.001

With all these findings, a prediction model with all original variables in logistic regression (herein referred to as full model) was created using area under the ROC curve (AUC) as benchmark (AUC of 0.934 and a Brier score of 0.0956). Next, after categorizing all variables, a prediction model based on the ranking of the OR with an AUC of 0.919 and a Brier score of 0.0971 was created. A sum score, termed AD score (ADS), was derived from the prediction model for risk stratification in view of its clinical utility in assisting prediction of malignant risk (Table 4). There is no significant differences between the 2 prediction model scores (see Fig. 4). To facilitate better understanding of the prediction model, pictorial case examples with clinical application of AD scores are demonstrated in Fig. 5 and Figs. 6 and 7 (see supplementary data).

**Table 4:** Architectural distortion score (ADS) and corresponding suggested BI-RADS category in predicting breast malignancy for AD lesions detected at digital breast tomosynthesis

Characteristic	Odds ratio	Weight	Rank/score
CEDM background parenchyma enhancement			
- Minimal	1.0	0	0
- Mild	1.0	1	1
- Moderate or marked	2.9	3	3
Size of AD on CEDM			
- < 0.7 cm	1	0	0
- ≥ 0.7 cm	1.8	2	2
Enhancing morphology#			
- Focus	NA	NA	6
- Mass/non-mass	NA	NA	7
Enhancement intensity			
- Absent	NA	0	0
- Mild	NA	1	1
- Moderate	1.7	2	2
- Marked	30.2	30	5
Enhancing spiculations of AD on CEDM			
- Present	13.9	14	4
- Absent	1.0	0	0
Age (≥ 52 years)	3.1	3	3

*AD* architectural distortion, *CEDM* contrast enhanced digital mammography

AD Score and corresponding suggested BI-RADS category

0–6 benign: 0% malignant (BI-RADS 2)

7–9 probably benign: up to 2% (BI-RADS 3)

10–14 low suspicious (> 2 to ≤ 10%) (BI-RADS 4A)

15–17 moderate suspicious (> 10 to ≤ 50%) (BI-RADS 4B)

≥ 18 highly suspicious (> 50%) (BI-RADS 4C, 5)

#No odds ratio available as there are zero counts. Given the score of highest rank as *p* < 0.001 in univariate analysis

**Fig. 5:**
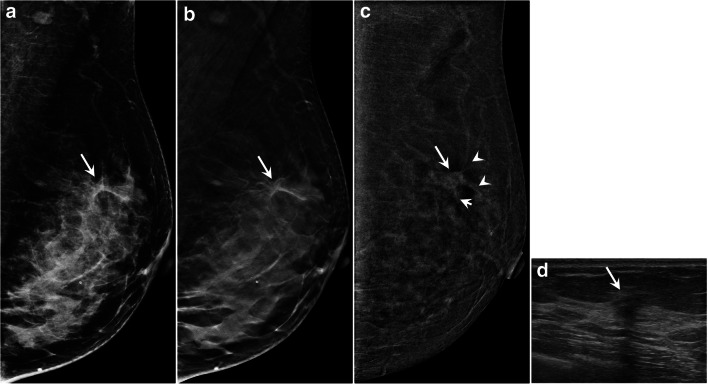
A 49-year-old woman undergoing screening mammography. Mediolateral oblique (MLO) view of full-field digital mammography (FFDM) (**a**) and digital breast tomosynthesis (DBT) (**b**) depicting an architectural distortion (arrows) in the left breast. MLO view of contrast-enhanced digital mammogram (CEDM) (**c**) shows a 1.2-cm enhancing lesion (arrows) with the following characteristics: mild background parenchyma enhancement (score 1); size of lesion > 0.7 cm (score 2); mass enhancement (score 7); moderate lesion enhancement (score 2); and presence of enhancing spiculations (score 4) (arrowheads). Architectural distortion score (ADS) is 16, and the lesion is categorized as BI-RADS 4B. (**d**) Correlative ultrasonographic image shows a hypoechoic mass (arrow) with posterior shadowing. Histopathology result: 0.8 cm invasive breast cancer

## Discussion

Many imaging modalities have been studied in the evaluation of AD, including DBT with or without ultrasound correlates [6, 7, 18–20] and MRI [11, 21], but each has its own limitation. With the increased detection of AD from growing use of DBT, there is an unmet clinical need for a cost-effective imaging modality for characterization of AD lesions to reduce the need for unnecessary invasive procedures such as biopsy or surgical excision. Our results demonstrated that CEDM can be a very useful adjunct tool in assessment of AD lesions. We found that AD lesion with no or mild contrast enhancement on CEDM has a lower chance of breast malignancy than that with moderate or marked contrast enhancement. The use of an accurate prediction model (AUC = 0.921, Brier score = 0.099) can assist in decision making during assessment of AD lesions according to BI-RADS categories for breast radiologists or surgeons (see Figs. 6 and 7 under Supplementary Data).

Several studies have demonstrated DBT-only AD to have a lower PPV for malignancy as compared to mammography-detected AD [5, 6]. This could partially explain the relatively low PPV of 48.5% on CEDM in our study as 22.3% of AD lesions were detected on DBT only. The relatively low PPV for AD lesions on CEDM also suggests that not all AD lesions which enhance on CEDM would warrant a biopsy or surgical excision to achieve histological diagnosis. The associations between characteristics of AD on CEDM and risk of malignancy were assessed using a multivariate analysis. This demonstrated 2 factors on CEDM which showed statistical significance: (1) marked enhancement intensity of AD on CEDM (rank score = 5); and (2) presence of enhancing spiculations on CEDM (rank score = 4).

Marked enhancement intensity of AD on CEDM has demonstrated a significant association with malignancy in our study. While many benign breast lesions enhance (e.g., mild hyperplasia or proliferative lesions without atypia), these usually only demonstrate mild enhancement on contrast-enhanced modalities such as MRI [22] as opposed to marked enhancement of malignant lesions due to underlying angiogenesis. In our study, marked enhancement on CEDM was a significant factor with an OR of 22.6 (95%CI 3.1, 166.6; *p* = .002). Of note, the assessment of enhancement intensity in our study remains subjective and further studies with use of quantitative measures or software may help to better stratify malignant risk in the future [23].

Secondly, spiculated margins of radiologically detected masses have been well-known morphologic criteria for breast malignancy [22, 24]. While this is largely unsurprising for masses, enhancing spiculations for AD have not been well described on CEDM. In our study, we investigated the association of enhancing spiculations on CEDM with risk of malignancy which demonstrated an OR of 9.1 (95%CI 2.2, 36.5; *p* = .002).

The presence of moderate to marked BPE has been well known to cause considerable effect in detection of breast malignancy on contrast-enhanced modalities such as MRI [25, 26]. Moderate to marked BPE could potentially mask underlying malignancies, resulting in difficult evaluation of target lesions. While our study did not demonstrate any significant association of malignancy, moderate to marked BPE (rank score = 3) remains a significant clinical factor and a potential pitfall while evaluating contrast enhancement of lesion on CEDM images.

Contrary to the relative low PPV, our results demonstrated an extremely high NPV of 100% in 26 (27.7%) of 94 lesions which showed no suspicious enhancement on CEDM. Bearing in mind that the moderate to marked BPE could mask underlying malignancies, the results suggest that the absence of AD lesion enhancement on CEDM on a background of minimal or mild BPE has a lower risk of breast malignancy. However, invasive procedures should be considered for any AD lesion with clinical symptoms or other imaging abnormalities despite a low AD score on CEDM.

Our study has a few limitations. Firstly, 10 (10.6%) of 94 patients had only clinical diagnosis of benign lesions without histological diagnosis. As mentioned in the “Results” section, these 10 patients had additional evaluation with breast MRI for very subtle AD lesions on DBT. The remaining 4 (40%) of 10 patients had no suspicious enhancement of AD on CEDM. The final BI-RADS was based on MRI assessment and DBT follow-up as there is no commercial DBT biopsy device in our early period of CEDM study. Fortuitously, all 10 patients were followed up for 2 years according to BI-RADS recommendation with no patient drop-out. These cases were shown to remain unchanged in AD size and morphology and were hence regarded as benign. Secondly, the sample size (*n* = 94) for this study may be limited by the availability of pure AD lesions at a single institution and the prediction model calculations were based on the Asian population at age of 30–69 years old which do not factor in clinical breast symptoms. This necessitates a larger study (i.e., multicenter study or systematic review) to corroborate our findings and to validate our prediction model in different populations and CEDM vendors. Thirdly, we did not include ultrasound imaging into the AD score. Breast ultrasound is well known for its high operator dependence, and 3D automated breast ultrasound seemed to have better performance in the detection of architectural distortion on the coronal plane [27]. Target ultrasound may help identify obvious mass lesions with spiculated margins and other non-calcified lesions on mammogram. However, the ADS and corresponding suggested BI-RADS lexicons can provide PPV prediction just based on mammographic features alone and reduce bias between operators. In this study, surgical results of high-risk lesions such as atypical ductal hyperplasia (ADH) and lobular carcinoma in situ (LCIS) were classified as benign cases. The management of these high-risk lesions has been debated among experts; annual mammography or MRI screening is often considered necessary [28]. Lastly, the study did not analyze inter-reader agreement for the presented CEDM descriptive characteristics. In this risk prediction model study, the authors grouped as many subjective descriptive characteristics together to reduce potential bias and inter-reader disagreements (e.g., moderate and marked BPE are grouped together). The objective was to make as many variables as binary as possible to assist in better interpretation of CEDM images and adaptation of the risk prediction model. However, assessment of inter-agreement analysis of AD score as a whole could be assessed in a larger prospective study in the future.

## Conclusion

The absence of suspicious enhancement of AD lesion at minimal or mild BPE indicates low risk of breast malignancy. For AD lesions which enhance, marked enhancement intensity and presence of enhancing spiculations are independent factors with highest associated risk of malignancy on multivariate analysis. The ADS as mentioned can be a useful tool in assessment of BI-RADS categories of AD lesion at CEDM.

## Electronic supplementary material

ESM 1 (DOCX 4644 kb)

## Abbreviations

AD: Architecture distortion
CEDM: Contrast-enhanced digital mammography
DBT: Digital breast tomosynthesis
FFDM: Full-field digital mammography
ADS: Architecture distortion score
