# Supplementary material for: Architecture distortion score (ADS) in malignancy risk stratification of architecture distortion on contrast-enhanced digital mammography
Source: Eur Radiol. 2020 Oct 30;31(5):2657–66. doi: 10.1007/s00330-020-07395-3 (PMC8043942; doi:10.1007/s00330-020-07395-3)

**SUPPLEMENTARY MATERIALS**

*Figure 6*

Fig 6. A 51-year-old woman undergoing screening mammogram. (A, B) Mediolateral oblique (MLO) view of full field digital mammography (FFDM) and digital breast tomosynthesis (DBT) depicting an architectural distortion (arrow) in the left breast. (C) MLO view of contrast-enhanced digital mammogram (CEDM) of AD lesion (arrow) shows the following characteristics: minimal background parenchyma enhancement (BPE)(score 0); size of lesion <0.7 cm (score 0); absence of AD enhancement (score 0); and absence of enhancing spiculations (score 0). Architectural distortion score (ADS) is 0, and the breast lesion is categorized as BI-RADS 2. (D) Correlative ultrasonographic image shows a hypoechoic mass (arrow) with posterior shadowing. Histopathology result: radial scar.

a
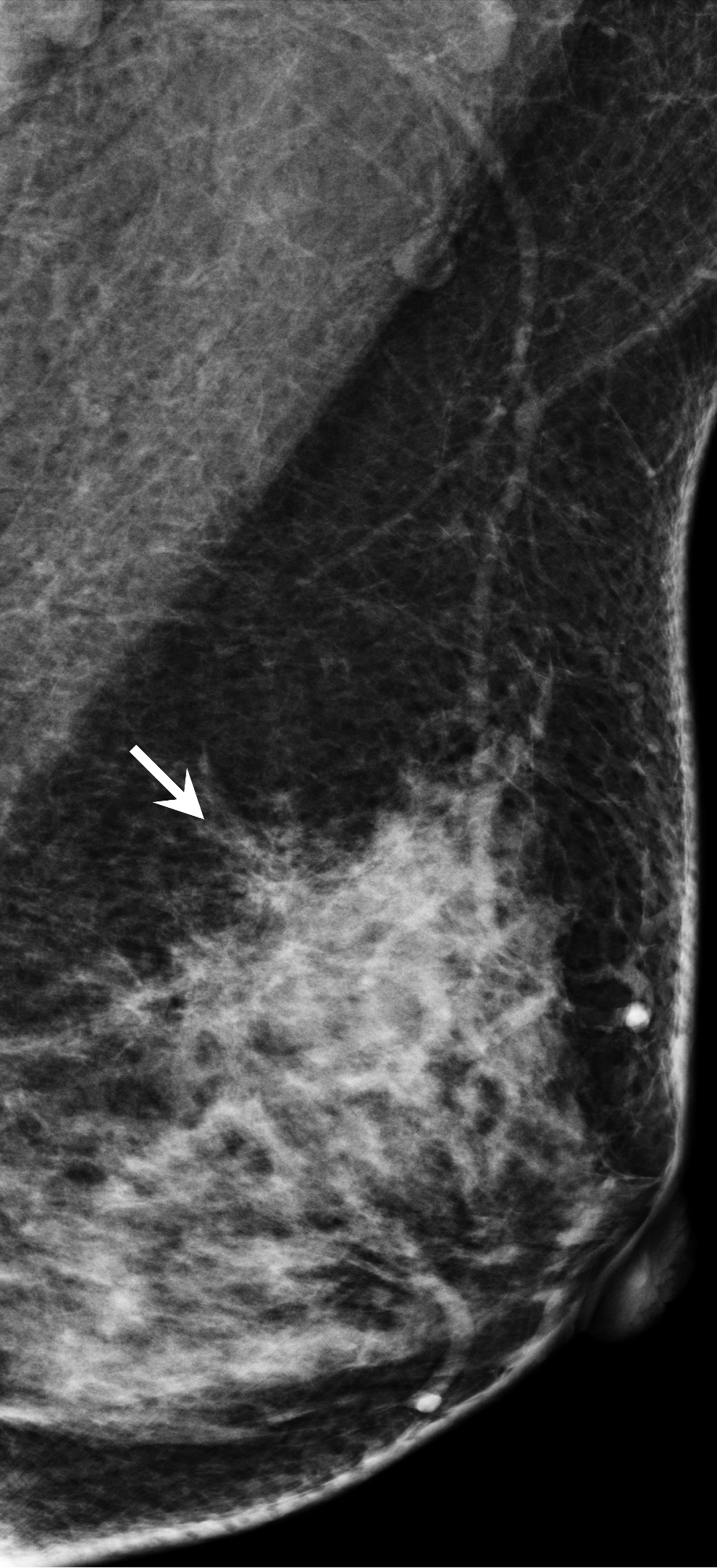


b
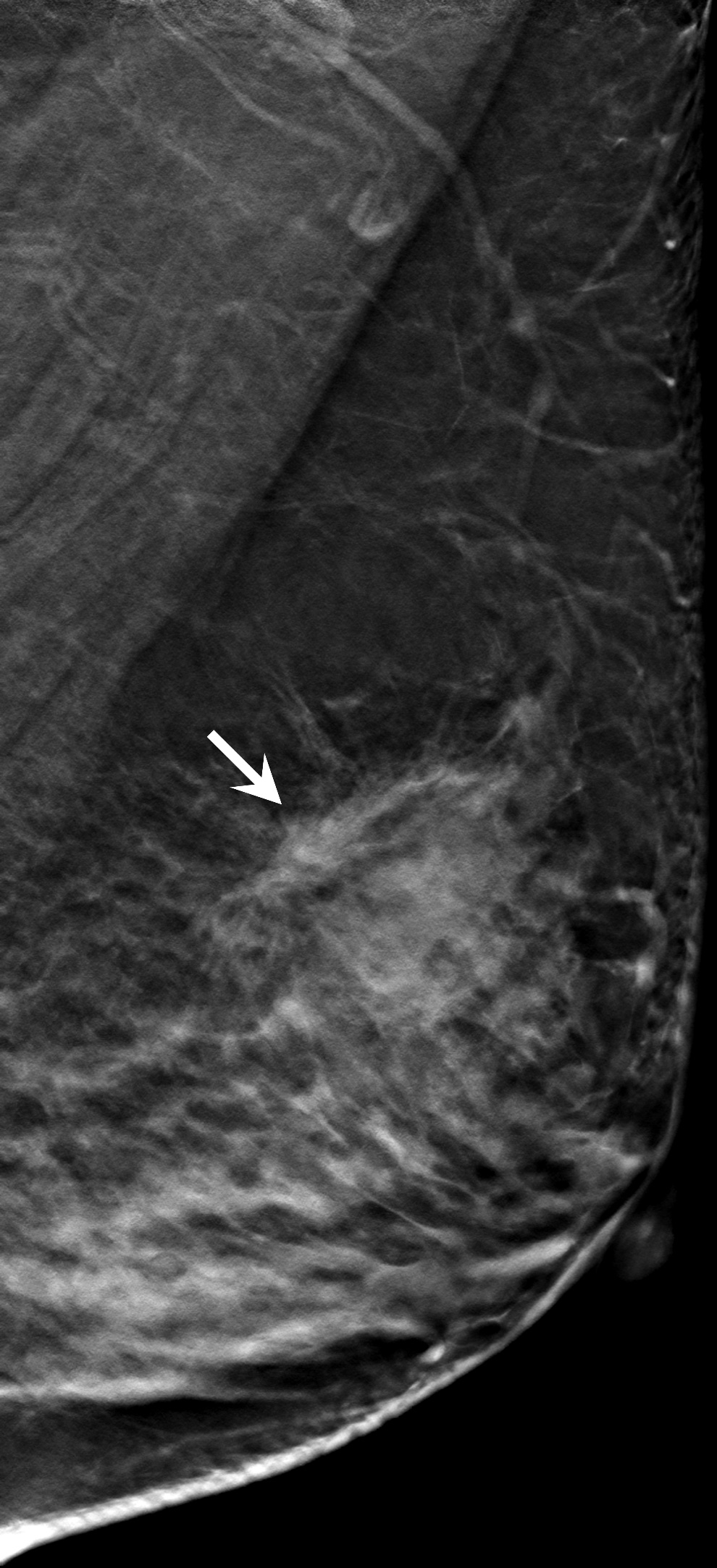


c
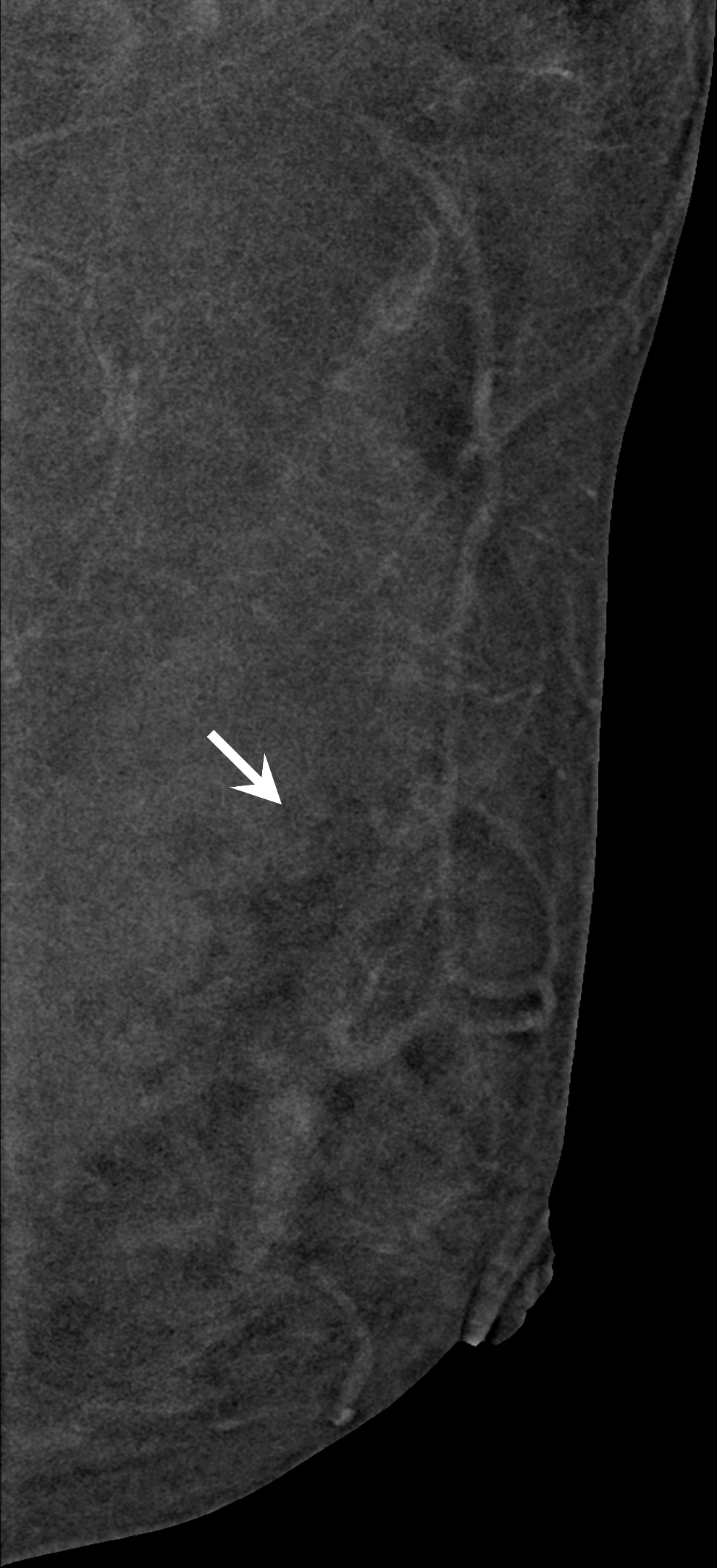


d
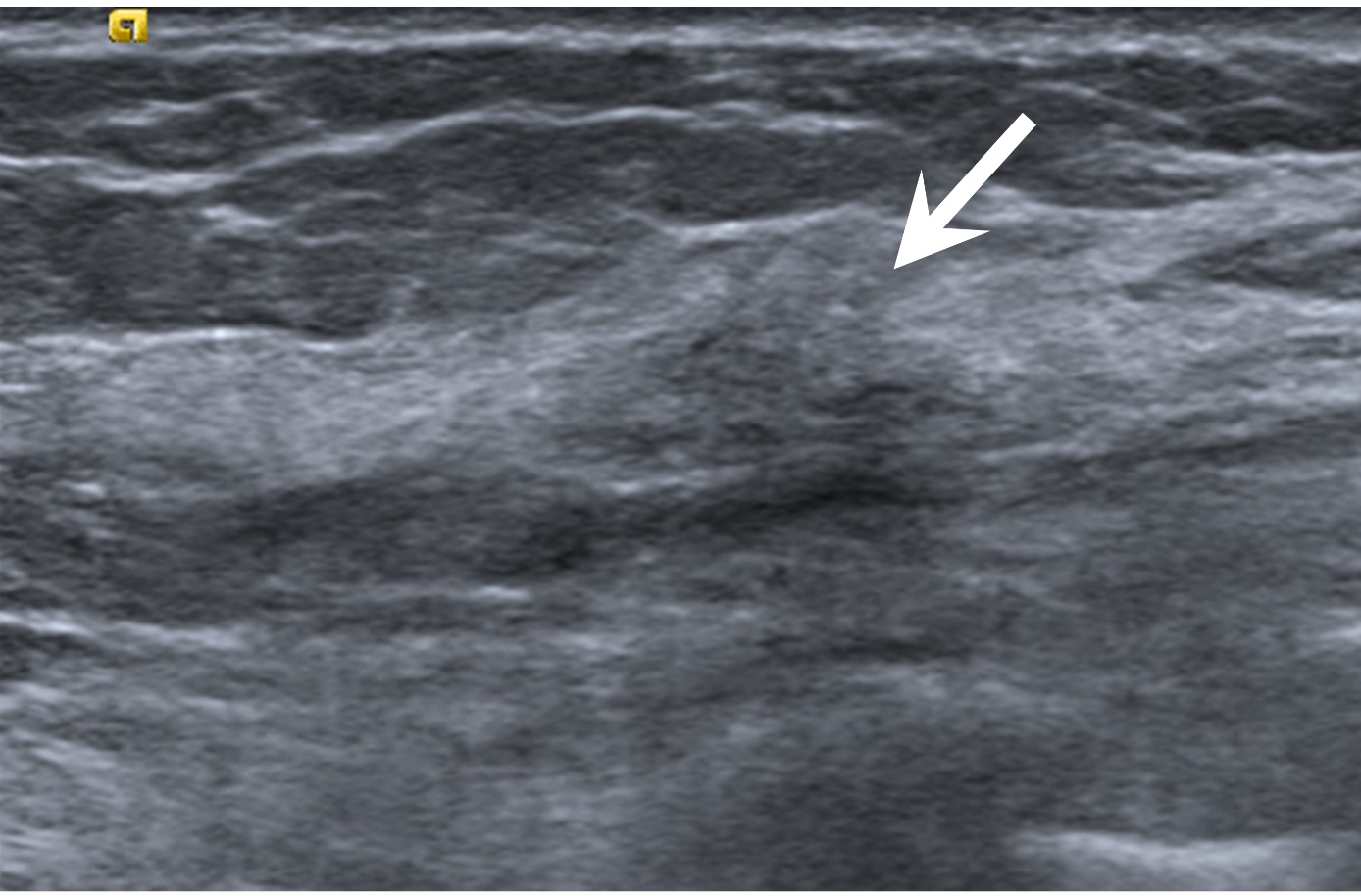


*Figure7*

Fig 7. A 68-year-old woman undergoing screening digital breast tomosynthesis (DBT). Mediolateral oblique (MLO) view of full field digital mammography (FFDM) (A) and digital breast tomosynthesis (DBT) (B) depicting an architectural distortion (arrows) in the right breast. MLO view of contrast-enhanced digital mammogram (CEDM) (C) shows a 0.5-cm enhancing lesion (arrow) with following characteristics: minimal background parenchyma enhancement (score 0); size of lesion <0.7cm (score 0); focus enhancement (score 6); mild lesion enhancement (score 1); absence of enhancing spiculations (score 0), and age ≥ 52 years (score 3). Architectural distortion score (ADS) is 10, and the lesion is categorized as BI-RADS 4A. (D) Correlative contrast-enhanced breast MRI shows an enhancing focus (arrow) at right breast corresponding region. Histopathology result: radial scar.

a
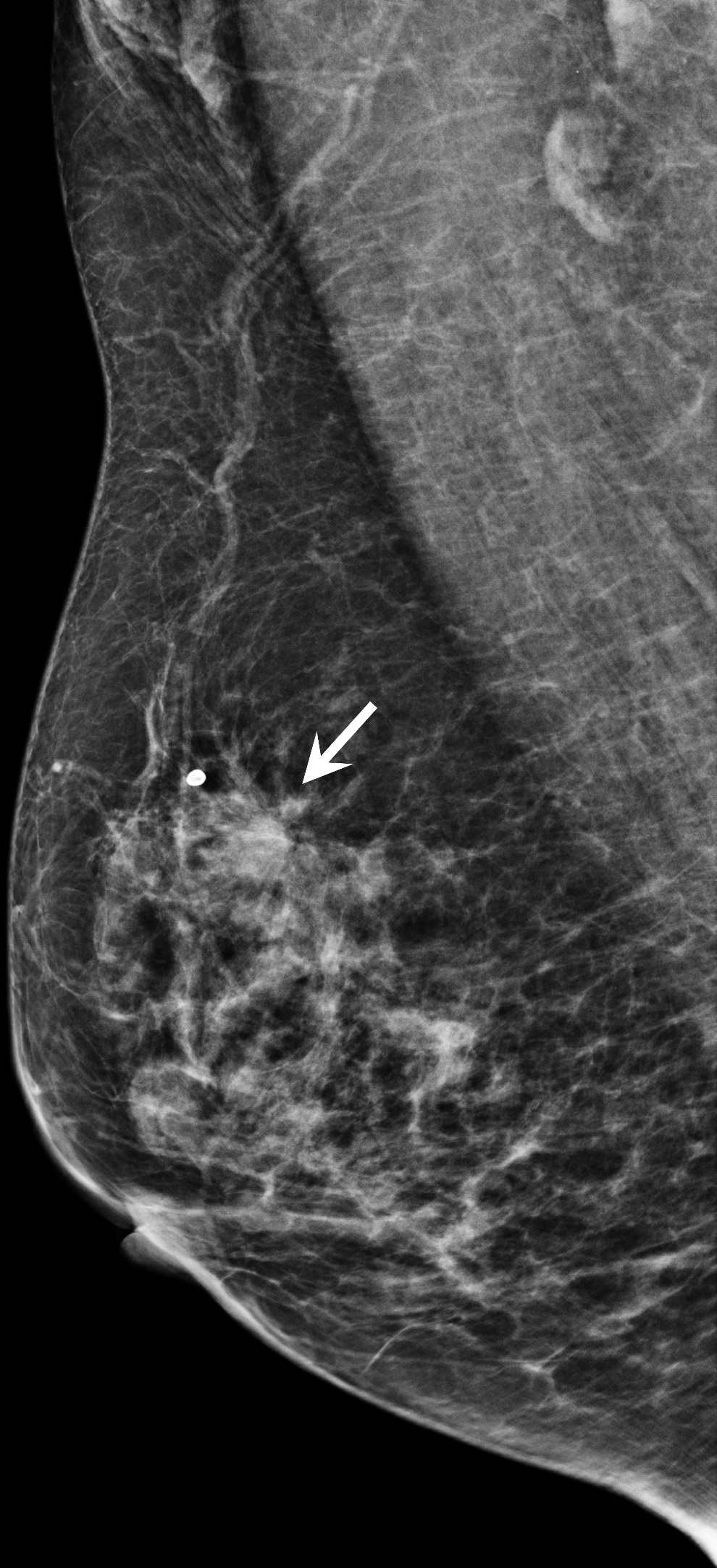


b
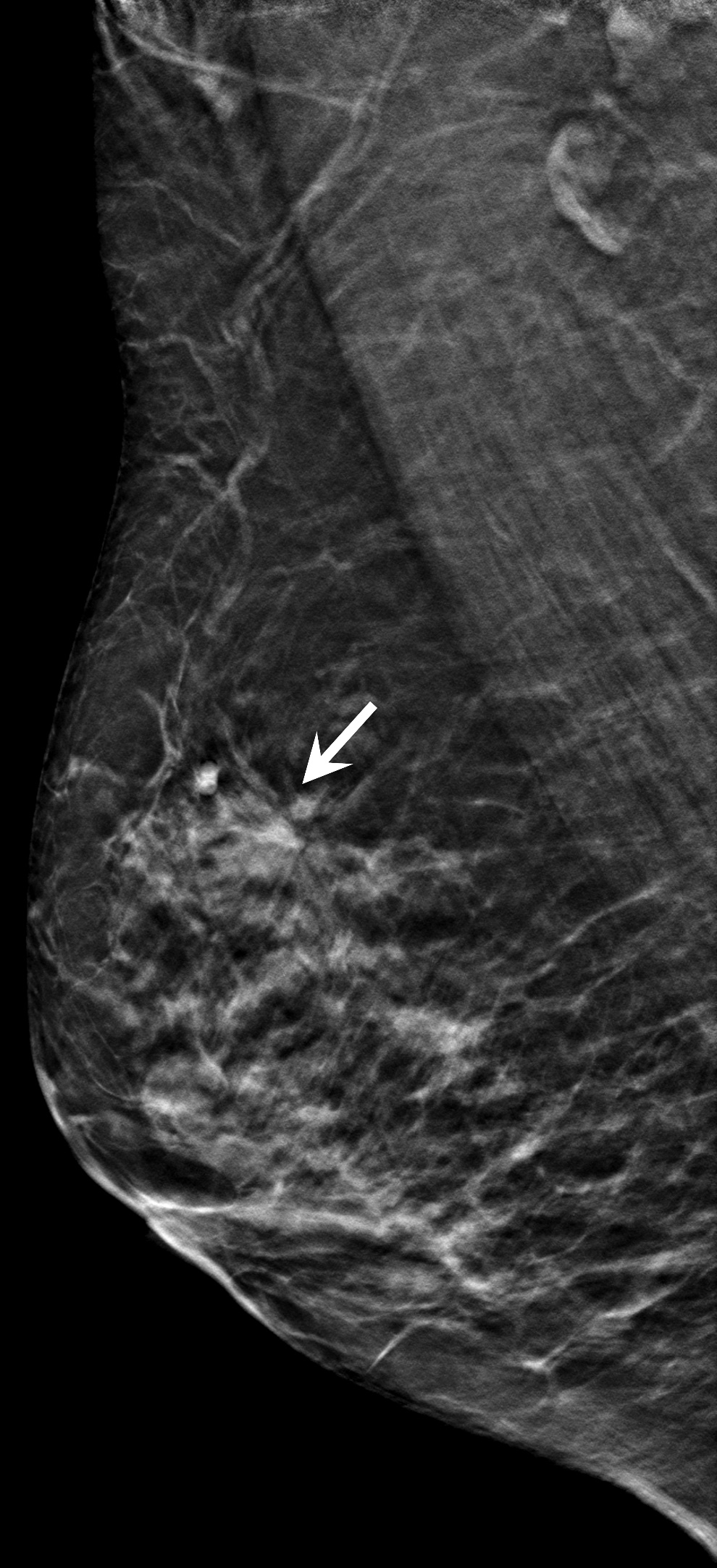


c
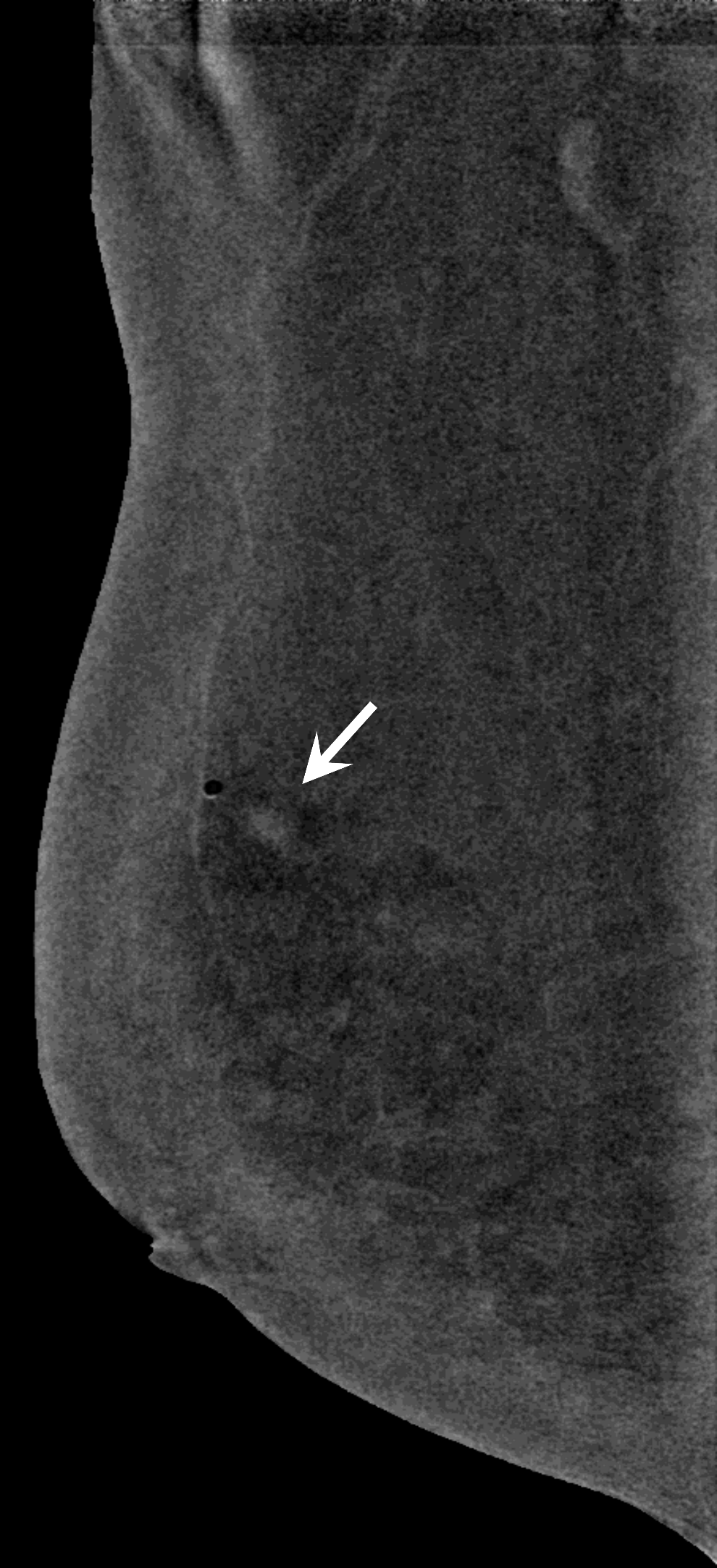


d
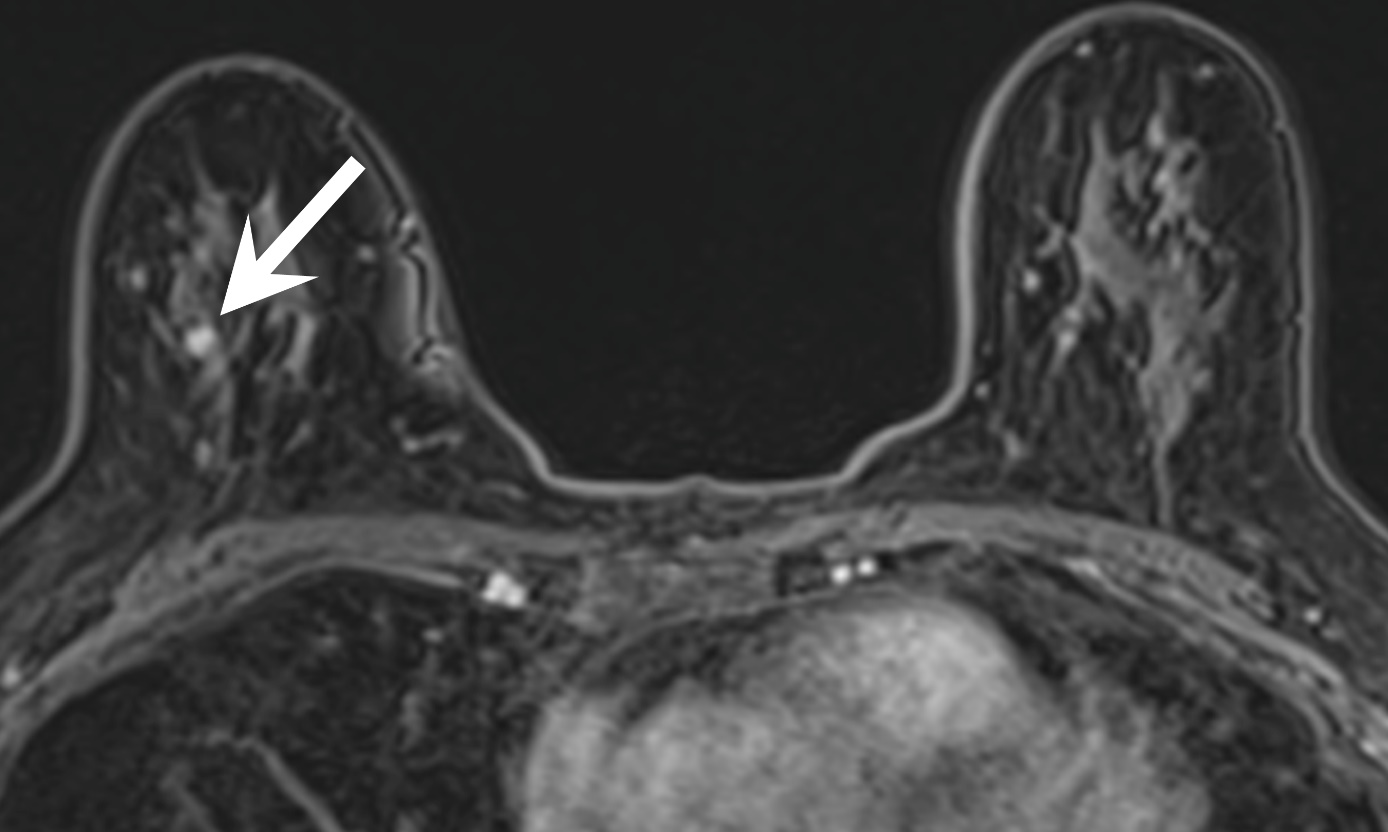

Supplement: Supplementary file 1 — (DOCX 4644 kb) [file 330_2020_7395_MOESM1_ESM.docx]
